# Optic canal decompression in severe therapy refractory optic neuritis: a case report

**DOI:** 10.1016/j.ajoc.2026.102626

**Published:** 2026-07-14

**Authors:** Armin Handzic, Pia Niederau, Caroline Seebauer, Christian P. Kamm, Oliver Job

**Affiliations:** a University of Lucerne, Cantonal Hospital of Lucerne, Department of Ophthalmology, Lucerne, Switzerland; b University of Lucerne, Cantonal Hospital of Lucerne, Department of Neuroradiology, Lucerne, Switzerland; c University of Lucerne, Cantonal Hospital of Lucerne, Department of Otorhinolaryngology Head and Neck Surgery, Lucerne, Switzerland; d University of Lucerne, Cantonal Hospital of Lucerne, Department of Neurology, Lucerne, Switzerland; e Department of Neurology, Inselspital Bern, University Hospital and University of Bern, Bern, Switzerland

**Keywords:** Optic neuritis, Multiple sclerosis, Canalicular compartment syndrome, Optic canal decompression, Case report

## Abstract

**Purpose:**

To describe a unique case of successful optic canal decompression in radiologically confirmed severe optic neuritis involving the optic canal.

**Observations:**

A 24-year-old woman experienced severe visual loss in the right eye due to retrobulbar optic neuritis (ON), as an initial symptom of multiple sclerosis. High-dose corticosteroids and subsequent plasmapheresis yielded no improvement. Thus, visual prognosis was considered poor. Since MRI demonstrated ON within the optic canal with only minimal contrast enhancement, findings were compatible with a canalicular compartment syndrome. As a result, an unconventional treatment approach with optic canal decompression (OCD) was pursued. Immediately after the intervention, the patient noticed improved color vision and continued visual improvement, with vision almost restored to 20/30 one month after the intervention.

**Conclusions and Importance:**

This case report demonstrates that OCD can be performed safely in severe ON and that it might represent a valuable treatment option in selected cases of ON.

## 1 Introduction

Severe therapy refractory optic neuritis (ON) is a rare phenomenon. Nevertheless, poor visual outcome occurs in 6% of patients according to the optic neuritis treatment trial.^1^ This landmark study further provided valuable information regarding the early course of visual recovery in ON. One important finding was that most treated patients demonstrated improvement of visual acuity of at least one line within two weeks.^2^ Our patient did not follow this typical course, thus poor visual outcome was expected. One plausible explanation was that an additional canalicular compartment syndrome occurred which induced ischemic damage to the optic nerve. Since radiological findings were present to support this consideration, we performed an unconventional treatment approach consisting of optic canal decompression (see Table 1).

**Table 1:** Timeline of clinical course, investigations and treatment.

Time point	Clinical course, investigations, and treatment
Day −2	Sudden painless visual loss in the right eye
Day 0	Referral for presumed central retinal artery occlusion. VA 20/100 OD, 20/20 OS; strong right RAPD; normal anterior and posterior segment examination; dense central scotoma on Octopus and Goldmann perimetry; normal OCT
Day 0–1	Retinal ischemia excluded; diagnosis of atypical right optic neuritis. MRI brain/orbits showed T2 hyperintensity with subtle contrast enhancement of the canalicular portion of the right optic nerve
Following days	CSF analysis and neuroimaging consistent with multiple sclerosis; MOG-IgG and AQP4 antibodies negative
Days 1–5	IV methylprednisolone 1 g/day for 5 days; no visual improvement, VA deteriorated to counting fingers
Subsequent days	Five cycles of plasmapheresis; no visual improvement
Day 14	Transnasal sphenoethmoidectomy with decompression of the bony optic canal, optic nerve sheath fenestration, and opening of the annulus of Zinn; IV immunoglobulins administered in parallel
Postoperative days 0–3	Immediate improvement in color vision; minimal RAPD and marked improvement of central scotoma
1 month	VA improved to 20/30; central scotoma resolved
6 months	Final VA 20/25

## 2 Case description

A 24-year-old woman was referred by her ophthalmologist to a tertiary center eye clinic for presumed central retinal artery occlusion. She presented with sudden painless visual loss in the right eye two days previous. She reported no recent infections and was otherwise healthy. On clinical examination, visual acuity (VA) was 20/100 for the right and 20/20 for the left eye. There was a strong right relative afferent pupillary defect (RAPD). Anterior and posterior segments were unremarkable. Octopus and Goldmann perimetry revealed a large, dense central scotoma (Fig. 1). Optic coherence tomography was normal. Given the lack of findings compatible with an ischemic retinal event, a diagnosis of right retrobulbar optic neuritis (ON) was made. The presence of painless and severe ON raised the suspicion of a canalicular involvement of the optic nerve which was confirmed by MRI brain/orbits with contrast and fat suppressed sequences (Fig. 2, Fig. 3). Of note, there was only very subtle contrast enhancement but clearly noticeable T2-hyperintensity within the canalicular portion of the right optic nerve. Neuroimaging and cerebrospinal fluid findings fulfilled the 2024 McDonald criteria for multiple sclerosis (MS). MRI demonstrated dissemination in space with characteristic demyelinating lesions involving the right optic nerve, periventricular and supratentorial regions, as well as the spinal cord. Dissemination in time was established by the coexistence of gadolinium-enhancing optic nerve lesion and non-enhancing CNS lesions and the presence of CSF-restricted oligoclonal IgG bands (type 2). Blood work was negative for MOG-IgG and AQP4 antibodies. Treatment with solumedrol 1g daily for five days was started with no visual improvement. On the contrary, VA deteriorated to counting fingers. Treatment was therefore escalated to five cycles of plasmapheresis; however, this also yielded no improvement two weeks after onset of symptoms. At this stage, the visual prognosis was considered poor. In light of radiological findings compatible with a canalicular compartment syndrome (CCS), the patient was offered an unconventional and experimental treatment approach consisting of optic canal decompression (OCD). The procedure was performed via a transnasal sphenoethmoidectomy with decompression of the bony optic canal, optic nerve sheath fenestration and opening of the annulus of Zinn. Owing to the unknown effectiveness of the surgical intervention, intravenous immunoglobulins (IVIG) were administered in parallel. After surgery, the patient immediately noticed improved color vision and at three days post-surgery, there was only minimal RAPD and the central scotoma had improved significantly. VA initially remained at counting fingers, but the patient demonstrated continuous visual improvement. One month after surgery, VA had improved to 20/30, and the central scotoma had disappeared. Six months post-surgery, final VA was 20/25. A summary of these events can be found in Table 1.

**Fig. 1:**
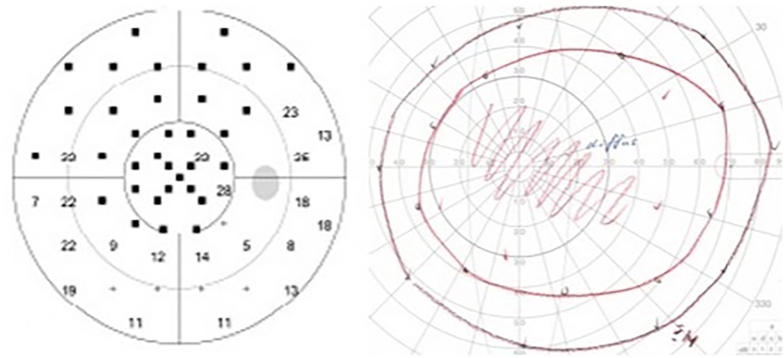
Octopus and Goldmann perimetry demonstrating a large, dense central scotoma.

**Fig. 2:**
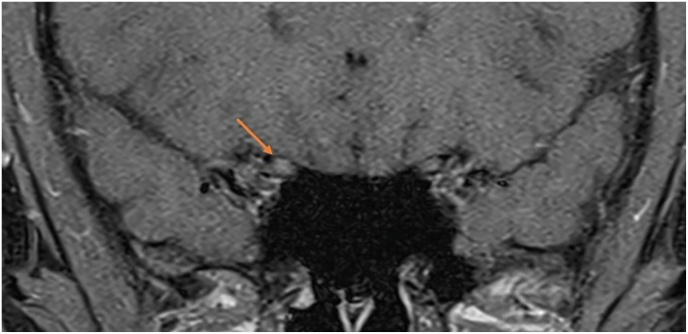
MRI orbits with T1 post contrast fat-saturated coronal sequences showing mild contrast enhancement of the right optic nerve within the optic canal (orange arrow). (For interpretation of the references to color in this figure legend, the reader is referred to the Web version of this article.)

**Fig. 3:**
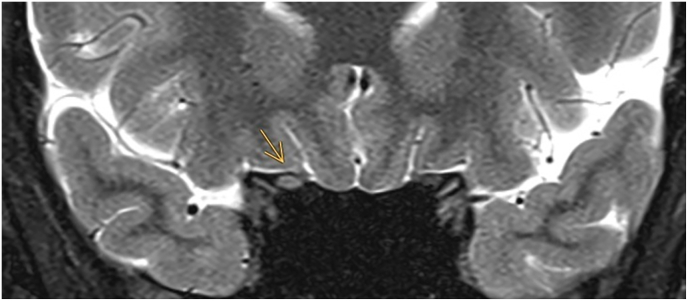
MRI orbits with T2 coronal sequences showing corresponding T2-hyperintensity of the right optic nerve within the optic canal (orange arrow). (For interpretation of the references to color in this figure legend, the reader is referred to the Web version of this article.)

## 3 Discussion

This case demonstrates the potential value of OCD in patients with severe therapy refractory ON involving the optic canal. The rationale behind our unconventional treatment approach was based on the hypothesis of a CCS producing additional ischemic damage to the optic nerve. Miller et al. (1988) first proposed the concept of a CCS to explain the association between poor visual outcomes and ON located within the optic canal, by assessing T2-sequences on MRI images.^3^ Handzic et al. added further support to this hypothesis by using contrast-enhanced sequences, showing that mild contrast enhancement on neuroimaging was associated with poor visual outcome.^4^ The rationale behind this study was that severe CCS would reduce blood perfusion to the optic nerve, and in turn, reduce contrast enhancement. Thus, since our case demonstrated subtle contrast enhancement within the optic canal that could have easily been misinterpreted as unremarkable neuroimaging, visual prognosis was considered poor. The patient was therefore offered an OCD to release the pressure within the optic canal and reduce the ischemic damage.

The poor visual prognosis in this case was further supported by findings from the Optic Neuritis Treatment Trial (ONTT).^1^^,^^2^ This landmark study provided detailed insight into the early course of visual recovery in ON with and without corticosteroid treatment. First, the most significant improvements in visual recovery were observed at days 4 and 15.^1^ More specifically, at least one line of visual acuity improvement occurred by the day 15 visit in 91.5% in patients treated with solumedrol who also demonstrated a faster recovery than those receiving placebo.^2^ Beck et al. therefore concluded that failure to achieve at least one line of visual acuity improvement within the first 3 weeks after symptom onset should be considered atypical.^2^ This was also the case in our patient. Consequently, the visual recovery observed in our patient did not align with the expected course of MS-associated ON. Second, at 6 months, 6% of patients in the solumedrol group had poor visual outcomes. More precisely, Chen et al. reported that in the ONTT, only 4 patients treated with intravenous corticosteroids remained count-finger vision at 2 weeks, and all experienced suboptimal outcomes: 1 improved to 20/80 whereas the remaining 3 had final visual acuity worse than 20/200.^5^ Since our patient had no visual improvement within two weeks of medical treatment, we were concerned that she would also experience poor visual outcome. Lastly, as the patient experienced immediate improvements in vision and visual function post-surgery, poor visual prognosis was supported, and spontaneous recovery was considered highly unlikely. These reasons support the fact that OCD was critical for visual recovery. A delayed effect of plasmapheresis cannot be completely ruled out as a contributing factor. However, Chen et al. found no significant difference in visual outcome in patients treated with plasmapheresis compared to the ONTT cohort.^5^ Thus, high-level evidence for plasmapheresis in ON is still lacking. A significant effect of IVIG is unlikely due to the final diagnosis of MS as there is no evidence for its efficacy in this setting.

Further supporting the existence of a CSS, as well as the potential benefit of OCD, is the neuroanatomical structure of the optic nerve. Hiraoka et al. demonstrated that the papillomacular bundle in primates retains its structure and runs through the central portion of the optic nerve.^6^ Since blood supply for the optic nerve derives from the pial plexus in a radial pattern, the very central part of the optic nerve has the lowest pressure for blood perfusion.^7^ As a result, this represents the most vulnerable location for ischemic injury in the setting of elevated pressure within the optic canal. Hence, a CCS would preferably produce a central scotoma as it was the case in our patient. Moreover, this could explain why VA took longer to recover.

In terms of surgery, optic canal decompression via a transnasal endoscopic approach represents a well-established minimally invasive surgical corridor that corresponds to standard endoscopic sinus and skull base procedures, avoiding external incisions, brain retraction, or orbital manipulation.^8^ Surgical principles are largely similar in traumatic and nontraumatic optic neuropathies, typically involving endoscopic sphenoethmoidectomy with decompression of the inferomedial optic canal.^8^^,^^9^ In traumatic optic neuropathy (TON), acute edema and secondary ischemia within the confined canal are considered the primary mechanisms of injury, and visual improvement rates after early endoscopic decompression have been reported in 45–82% of cases.^9^^,^^10^ In contrast, inflammatory optic neuropathies develop subacutely, which may explain why OCD was still effective even after two weeks of symptoms. However, the upper limit of the therapeutic window remains to be determined in a larger case series. Our surgical approach was conceptually similar to decompression techniques described for traumatic optic neuropathies, since we believe both entities may share a similar pathophysiological mechanism. Reported complications include cerebrospinal fluid leak, postoperative epistaxis, and sinonasal morbidity, which are generally uncommon in experienced hands.^8^

In conclusion, this case illustrates the use of OCD in the setting of radiologically confirmed canalicular ON and highlights the potential value of OCD in cases with severe therapy refractory ON involving the optic canal and, as a result, supports the existence of a CCS. It also demonstrates that OCD can be achieved over a safe and minimally invasive surgical intervention. Thus, OCD may become a valuable expansion of our treatment armamentarium in selected cases of ON, but controlled trials are needed to corroborate the potential benefit.

## 4 Patient perspective

One evening, I noticed that colors appeared less vivid in my right eye. By the next morning, vision had deteriorated to the point where I could only perceive outlines, which was deeply distressing. I was unable to read at work and sought immediate ophthalmologic evaluation. Despite rest, my vision rapidly worsened, and by the following day I was almost completely blind in the affected eye.

I was urgently referred to a tertiary eye clinic with suspected inflammation of the optic nerve. Extensive investigations followed, including neurological assessment, lumbar puncture and MRI. The findings suggested an autoimmune optic neuritis, and multiple sclerosis was considered the most likely diagnosis. Receiving this information was emotionally overwhelming.

High-dose intravenous corticosteroids were administered, followed by plasmapheresis due to continued visual decline; however, no improvement was observed. I was informed that visual recovery was unlikely, which left me feeling hopeless and devastated.

As a final option, a neuro-ophthalmologist proposed an experimental optic canal decompression based on MRI evidence of optic nerve compression. At this point in time, I was emotionally completely overwhelmed but did not lose hope. Despite uncertainty regarding its efficacy, I agreed to the procedure. Remarkably, on the evening after surgery, I was able to perceive color again for the first time, which restored hope.

The postoperative course was uncomplicated, apart from transient nasal pressure and headaches. Over subsequent follow-up, my vision continued to improve. Although it has not fully returned to baseline, I am now able to live independently and cope well with my diagnosis of multiple sclerosis. Today, I know that I made the right decision and that the surgical intervention has helped restore my vision. Thus, I am deeply grateful to the treating team and hopeful that my courage in pursuing an experimental treatment approach may benefit other patients in the future.

## Ethical considerations

Ethics approval was not required for this case report.

## Consent to participate

Not applicable.

## Consent for publication

Patient consent was obtained and is on file.

## Artificial intelligence statement

Artificial intelligence has not been used to create this manuscript.

## Funding statement

No funding was received for this work.

## CRediT authorship contribution statement

**Armin Handzic:** Conceptualization, Writing – original draft. **Pia Niederau:** Writing – review & editing. **Caroline Seebauer:** Writing – review & editing. **Christian P. Kamm:** Writing – review & editing. **Oliver Job:** Writing – review & editing.

## Declaration of competing interest

The authors declare that they have no known competing financial interests or personal relationships that could have appeared to influence the work reported in this paper.

## Data Availability

Not applicable.
